# Association of methylenetetrahydrofolate reductase ( *MTHFR* ) gene polymorphisms (C677T and A1298C) with thyroid dysfunction: A meta-analysis and trial sequential analysis

**DOI:** 10.20945/2359-3997000000471

**Published:** 2022-06-27

**Authors:** Rui Yang, Danhua Pu, Rongrong Tan, Jie Wu

**Affiliations:** 1 The First Affiliated Hospital of Nanjing Medical University Department of Obstetrics and Gynecology State Key Laboratory of Reproductive Medicine Nanjing China State Key Laboratory of Reproductive Medicine, Department of Obstetrics and Gynecology, The First Affiliated Hospital of Nanjing Medical University, Jiangsu Province Hospital, Jiangsu Women and Children Health Hospital, Nanjing, China

**Keywords:** Thyroid dysfunction, methylenetetrahydrofolate reductase ( *MTHFR* ), polymorphism, risk

## Abstract

Recent studies have shown that two common methylenetetrahydrofolate reductase ( *MTHFR* ) gene polymorphisms (C677T and A1298C) might correlate with thyroid dysfunction, but the results remain inconsistent. We carried out a meta-analysis aiming to assess the relationship of both polymorphisms with thyroid dysfunction. The PubMed, EMBASE, CNKI (China National Knowledge Infrastructure), CBMdisc (China Biology Medicine disc), WeiPu and Wanfang databases were searched up to September 2021. Case-control and cohort studies on *MTHFR* polymorphism and thyroid dysfunction were identified. Eight studies from six publications were finally included in our meta-analysis, including 817 patients and 566 controls. After pooled analysis, we found that the *MTHFR* C677T polymorphism was associated with an increased risk of hypothyroidism (TT vs. CC+CT/recessive model: OR = 2.07, 95% CI: 1.02-4.20, P = 0.04; TT vs. CC/homozygote model: OR = 2.35, 95% CI: 1.13-4.86, P = 0.02), while trial sequential analysis (TSA) revealed that it could be a false positive result. The *MTHFR* A1298C polymorphism was related to a decreased risk of hypothyroidism (C vs. A/allele model: OR = 0.63, 95% CI: 0.44-0.92, P = 0.02; CC vs. AC+AA/recessive model: OR = 0.42, 95% CI: 0.22-0.79, P = 0.007; CC vs. AA/homozygote model: OR = 0.43, 95% CI: 0.25-0.85, P = 0.02), which was conclusive according to TSA. The results of this meta-analysis suggest that *MTHFR* A1298C seems to be a protective factor for hypothyroidism, while the *MTHFR* C677T polymorphism may be a risk factor. However, more well-designed studies with larger sample sizes are needed to obtain more reliable results of the association between the *MTHFR* C677T polymorphism and hypothyroidism.

## INTRODUCTION

Thyroid dysfunction is a common endocrine disorder that always results from autoimmune thyroid diseases (AITDs), such as Hashimoto’s thyroiditis (HT) and Graves’ disease (GD). AITDs can affect people at any age, but women of reproductive age (30~50 years old) are more likely to suffer from these diseases ( 1 , 2 ). Thyroid dysfunction is associated with several adverse perinatal outcomes in the mother and fetus, including infertility, miscarriage, hypertensive disorders, premature delivery, and decreased IQ in the offspring ( 3 , 4 ). Recently, several clinical and epidemiological studies have shown a relationship between methylenetetrahydrofolate reductase ( *MTHFR* ) polymorphisms and thyroid dysfunction ( 5 - 7 ). One of the publications reported that two women diagnosed with Hashimoto’s thyroiditis suffered from infertility, and in both, *MTHFR* gene polymorphisms were identified ( 7 ).

Methylenetetrahydrofolate reductase (MTHFR) is a key enzyme in folate metabolism that catalyzes the irreversible conversion of 5, 10-methylenetetrahydrofolate to 5-methyltetrahydrofolate. MTHFR plays a crucial role in the regulation of DNA synthesis, the methylation cycle, and homocysteine concentrations in the blood ( 5 ).The *MTHFR* gene has been mapped to chromosomal region 1p36.22 and consists of 12 exons, encoding the 656-amino-acid protein.

C677T (rs1801133) and A1298C (rs1801131) are the two most common single nucleotide polymorphisms (SNPs) in the *MTHFR* gene ( 8 , 9 ). A C to T substitution at the 677th nucleotide of the *MTHFR* gene converts an alanine to a valine and causes thermolability of MTHFR ( 8 ). The *MTHFR* A1298C polymorphism results in a glutamic acid-to-alanine substitution leading to lower MTHFR enzyme activity than wild-type ( 10 ). A large body of literature has reported that genetic variation in this gene is associated with many diseases, such as neural tube defects, Alzheimer’s disease, vascular diseases and some kinds of cancer ( 11 - 17 ). Moreover, several studies have indicated that patients with hypothyroidism have elevated serum total homocysteine ( 18 - 26 ), which seemed to implicate the underlying correlation between the *MTHFR* gene polymorphism and hypothyroidism.

To date, several studies ( 5 , 6 , 27 - 32 ) have been carried out to explore the potential association between *MTHFR* gene polymorphisms and thyroid diseases (HT, GD, and subclinical/overt hyper- and hypothyroidism), but the results remain controversial. Here, we conducted a meta-analysis of all case-control and cohort studies to shed some light on the association between the *MTHFR* C677T and A1298C polymorphisms and thyroid dysfunction.

## MATERIALS AND METHODS

### Search strategy

PubMed (1950-2021), EMBASE (1974-2021) and Chinese databases, including the China National Knowledge Infrastructure (CNKI), China Biology Medicine disc (CBMdisc), WeiPu and Wanfang databases, were searched up to September 2021. Two authors independently performed a comprehensive literature search for relevant studies using the following terms: (“Methylenetetrahydrofolate reductase” OR “MTHFR”) AND (“thyroid” OR “thyroid diseases” OR “thyroid dysfunction” OR “hyperthyroidism” OR “hypothyroidism” OR “autoimmune thyroid disease” OR “Graves’ disease” OR “Hashimoto’s thyroiditis”). All references cited in the included studies or relevant reviews were also hand searched to identify any additional articles.

### Inclusion and exclusion criteria

Eligible studies included in the meta-analysis met the following inclusion criteria: ( 1 ) estimated the association between the *MTHFR* C677T and/or A1298C polymorphism and thyroid dysfunction (subclinical or overt hypo- and/or hyperthyroidism) or autoimmune thyroid diseases (Graves’ disease and/or Hashimoto’s thyroiditis); ( 2 ) were case-control or cohort studies; and ( 3 ) provided enough information on the frequency of genotypes in cases and controls. The exclusion criteria were as follows: ( 1 ) review articles, animal studies, simple commentaries, case reports, or unpublished reports and ( 2 ) reports containing no usable data.

### Quality evaluation and data extraction

The quality of the included studies was evaluated according to the Newcastle-Ottawa Scale (NOS), and only studies with a quality score of 6 or better were included for further analyses. Two authors independently extracted data from all eligible studies. Disagreement was settled by discussions. For each of the included studies, the following data were collected: the first author’s last name, publication year, country, ethnicity, thyroid function and sample size of cases and controls. Hardy-Weinberg equilibrium (HWE) in the controls was also performed as another reference to determine the quality of eligible studies.

### Statistical analysis

The associations between the *MTHFR* C677T and/or A1298C polymorphism and thyroid diseases were assessed by calculating the pooled odds ratios (ORs) and 95% confidence intervals (95% CIs). The statistical significance of the summary OR was determined with the Z-test. Five models, including the allele model (C677T: T *vs.* C; A1298C: C *vs.* A), dominant model (C677T: TT+TC *vs.* CC; A1298C: CC+AC *vs.* AA), recessive model (C677T: TT *vs.* TC+CC; A1298C: CC *vs.* AC+AA), homozygote model (C677T: TT *vs.* CC; A1298C: CC *vs.* AA) and heterozygote model (C677T: TC *vs.* CC; A1298C: AC *vs.* AA), were compared. The heterogeneity across the studies was estimated by the chi-square-based Q statistic test and the I^2^ test. The fixed-effect model (using the Mantel-Haenszel method) was used if I^2^ ≤ 50%; otherwise, the random-effect model (using the DerSimonian-Laird method) was applied. Subgroup analyses were performed by ethnicity and thyroid function. The Revman 5.3 (Review Manager Version 5.3. Copenhagen: The Nordic Cochrane Centre, The Cochrane Collaboration, 2014) was used for all statistical analyses. Stata software (version 16.0; Stata Corp LP, College Station, TX, USA) was used to analyze publication bias and for sensitivity analysis. Publication bias was investigated with Begg’s funnel plot, and funnel plot asymmetry was further assessed by Egger’s linear regression test ( 33 ). The significance of the intercept was determined by the t-test, and a P-value less than 0.05 was considered statistically significant. Sensitivity analysis was conducted by removing each individual study sequentially from the analysis to examine the effect of a single study on the collective results.

### Trial sequential analysis

TSA 0.9.5.10 software (http://www.ctu.dk/tsa/) was used for trial sequential analysis to minimize the type-I error and random error ( 34 ). The required information size (RIS) was determined based on a 5% risk of type I error, an 80% power of the study, and a case-control event proportion calculated from meta-analysis by the weighted average. The O’Brien-Fleming boundary or futility boundary was constructed to determine whether the present meta-analysis was sufficiently powered and conclusive. If the Z-curve crosses the TSA boundaries or futility area, there is sufficient information to support the conclusions, and further trials are unlikely to change the findings. If the Z-curve does not cross any of the boundaries or reach the RIS, the evidence is insufficient to make a firm conclusion. TSA would be conducted in the allele model. Meta-analysis, which presented a significant result in the pooled analysis, was also tested under TSA.

## RESULTS

### Study selection

**Figure 1:**
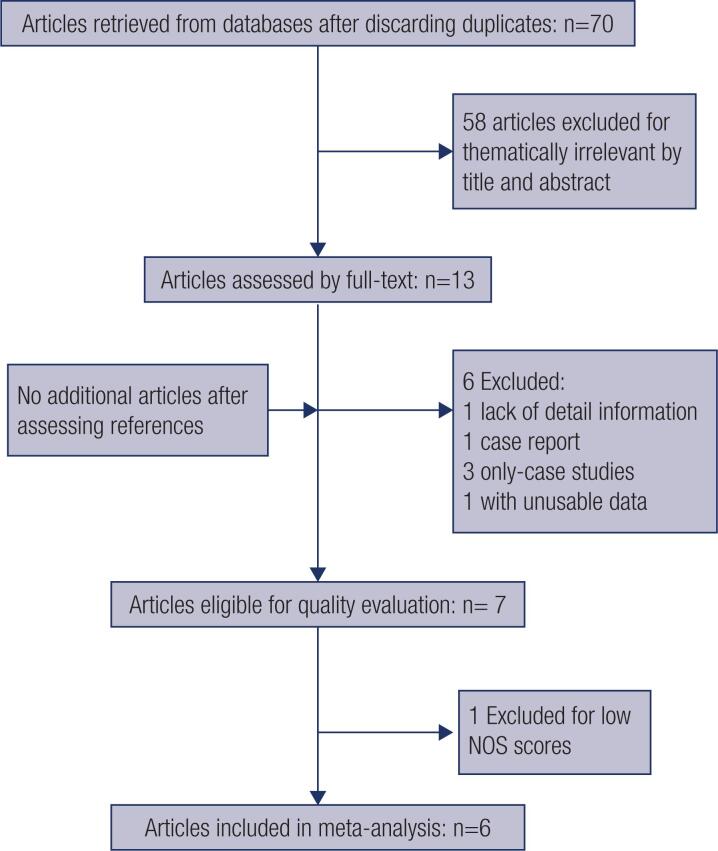
Flow chart of the study selection process.

Relevant citations were retrieved and preliminarily screened. Seventy studies were identified after discarding duplicates, and fifty-eight were excluded because they were thematically irrelevant based on the title and abstract. Thus, the full text of thirteen studies was searched and assessed. One conference abstract ( 35 ) was excluded due to a lack of detailed information. A case report ( 7 ) and three ( 20 , 27 , 29 ) case-only studies were deleted. A case-control study was excluded because the data were unusable ( 36 ). Finally, a total of seven papers ( 5 , 6 , 28 , 30 - 32 , 37 ) were eligible for quality evaluation ( Table S1 ), but one publication ( 28 ) was excluded for its low NOS scores. Therefore, six publications ( 5 , 6 , 30 - 32 , 37 ) were eligible for data extraction, two ( 6 , 31 ) of which contained two separate studies. Therefore, eight studies from six publications were ultimately included in this meta-analysis ( Figure 1 ).

In the present meta-analysis, eight studies met our criteria for *MTHFR* C677T polymorphism meta-analysis, and seven studies were eligible for *MTHFR* A1298C polymorphism meta-analysis. All the included studies were divided into “hyperthyroidism”, “hypothyroidism” or “not applicable (NA)” groups according to the thyroid function of the cases. The distributions of the *MTHFR* C677T and A1298C genotypes and the HWE of the included studies are shown in Table 1 .

**Table 1:** The characteristic of eligible studies in the meta-analysis

Studies	Country	Ethnicity	Types	Thyroid function	Cases			Controls			P_HWE_
					CC	CT	TT	CC	CT	TT	
( *MTHFR* C677T)											
Mao *et al.* , 2010	China	Asian	Graves’ disease	hyperthyroidism	51	88	60	36	131	68	0.0371
Arakawa *et al.* , 2012	Japan	Asian	Hashimoto’s disease	NA	45	63	11	36	35	13	0.3636
			Graves’ disease	NA	54	79	27				
			Hashimoto’s disease and Graves’ disease		99	142	38				
Lee *et al.* , 2016	Korea	Asian	Graves’ disease	hyperthyroidism	35	55	32	35	53	12	0.2332
Kvaratskhelia *et al.* , 2017	Georgia	Caucasian	Subclinical hypothyroidism	hypothyroidism	8	6	5	14	4	1	0.3638
Abu-Hassan *et al.* , 2019	Jordan	Caucasian	Hypothyroidism	hypothyroidism	49	32	17	55	32	12	0.0432
			Hyperthyroidism	hyperthyroidism	51	10	5				
			Hypothyroidism and Hyperthyroidism		100	42	22				
Kvaratskhelia *et al.* , 2020	Georgia	Caucasian	hypothyroidism	hypothyroidism	16	15	3	25	4	0	0.6900
				Total		817			566		
( *MTHFR* A1298C)					**AA**	**AC**	**CC**	**AA**	**AC**	**CC**	
Mao *et al.* , 2010	China	Asian	Graves’ disease	hyperthyroidism	139	41	6	178	55	2	0.3115
Arakawa *et al.* , 2012	Japan	Asian	Hashimoto’s disease	NA	75	36	3	37	26	1	0.1314
			Graves’ disease	NA	95	60	2				
			Hashimoto’s disease and Graves’ disease		170	96	5				
Lee *et al.* , 2016	Korea	Asian	Graves’ disease	hyperthyroidism	88	34	0	72	26	2	0.8445
Abu-Hassan *et al.* , 2019	Jordan	Caucasian	Hypothyroidism	hypothyroidism	45	37	16	37	29	32	0.0001
			Hyperthyroidism	hyperthyroidism	6	46	14				
			Hypothyroidism and Hyperthyroidism		51	83	30				
Kvaratskhelia *et al.* , 2020	Georgia	Caucasian	hypothyroidism	hypothyroidism	26	6	2	22	4	3	0.0054
				Total		777			526		

NA: not applicable; PHWE: p value of Hardy-Weinberg equilibrium.

### Meta-analysis of the C677T polymorphism

**Figure 2:**
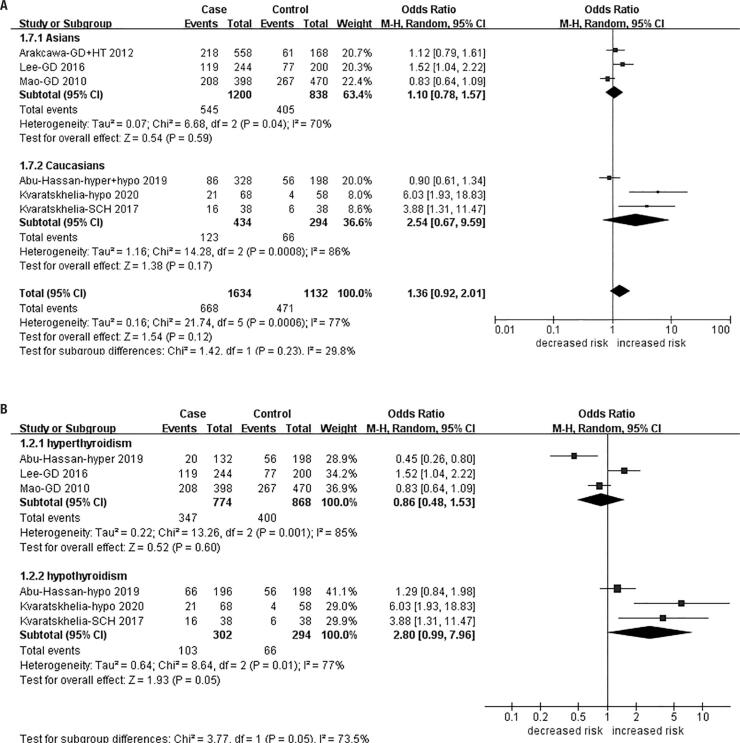
Association between the *MTHFR* C677T polymorphism and hypothyroidism risk (allele model: T *vs.* C). **A** Total analysis and subgroup analyses stratified by ethnicity; **B** Subgroup analyses stratified by thyroid function.

As shown in Table 2 , the pooled data of six publications indicated no association between the *MTHFR* C677T polymorphism and thyroid diseases in any of the five comparison models. However, subgroup analyses stratified by thyroid function showed a significant association of the *MTHFR* C677T polymorphism with hypothyroidism (TT *vs.* CC+CT/recessive model: OR = 2.07, 95% CI: 1.02-4.20, P = 0.04; TT *vs.* CC/homozygote model: OR = 2.35, 95% CI: 1.13-4.86, P = 0.02) ( Figure 2B and Supplemental Figures ). No relationship was found between the *MTHFR* C677T polymorphism and hyperthyroidism, even when reanalyzed by ethnicity ( Table 2 , Figure 2A and Supplemental Figures ).

**Table 2:** Meta-analysis of the association between *MTHFR* C677T polymorphism and thyroid disorders

Diseases	Comparison models	Studies/publications	I^2^	Model	OR (95% CI)	P
Thyroid disorders	Overall					
	T *vs.* C	8/6	77%	Random	1.36 (0.92, 2.01)	0.12
	TT+CT *vs.* CC	8/6	79%	Random	1.34 (0.76, 2.39)	0.31
	TT *vs.* CC+CT	8/6	44%	Fixed	1.29 (0.97, 1.71)	0.08
	TT *vs.* CC	8/6	64%	Random	1.40 (0.72, 2.71)	0.32
	CT *vs.* CC	8/6	76%	Random	1.17 (0.65, 2.08)	0.60
	Asians					
	T *vs.* C	4/3	70%	Random	1.10 (0.78, 1.57)	0.59
	TT+CT *vs.* CC	4/3	79%	Random	0.98 (0.51, 1.85)	0.94
	TT *vs.* CC+CT	4/3	65%	Random	1.29 (0.72, 2.31)	0.39
	TT *vs.* CC	4/3	76%	Random	1.16 (0.51, 2.65)	0.73
	CT *vs.* CC	4/3	79%	Random	0.89 (0.45, 1.78)	0.75
	Caucasians					
	T *vs.* C	4/3	86%	Random	2.54 (0.67, 9.59)	0.17
	TT+CT *vs.* CC	4/3	85%	Random	2.54 (0.56, 11.48)	0.22
	TT *vs.* CC+CT	4/3	36%	Fixed	1.58 (0.81, 3.10)	0.18
	TT *vs.* CC	4/3	60%	Random	3.09 (0.53, 18.16)	0.21
	CT *vs.* CC	4/3	80%	Random	2.03 (0.49, 8.41)	0.33
Hyperthyroidism	Overall					
	T *vs.* C	3/3	85%	Random	0.86 (0.48, 1.53)	0.60
	TT+CT *vs.* CC	3/3	79%	Random	0.65 (0.31, 1.33)	0.24
	TT *vs.* CC+CT	3/3	68%	Random	1.25 (0.60, 2.59)	0.55
	TT *vs.* CC	3/3	80%	Random	0.93 (0.33, 2.62)	0.88
	CT *vs.* CC	3/3	66%	Random	0.57 (0.30, 1.06)	0.07
	Asians					
	T *vs.* C	2/2	85%	Random	1.11 (0.61, 2.00)	0.73
	TT+CT *vs.* CC	2/2	84%	Random	0.83 (0.33, 2.07)	0.69
	TT *vs.* CC+CT	2/2	78%	Random	1.58 (0.66, 3.80)	0.31
	TT *vs.* CC	2/2	88%	Random	1.25 (0.30, 5.19)	0.76
	CT *vs.* CC	2/2	74%	Random	0.69 (0.32, 1.48)	0.34
Hypothyroidism	Overall (all Caucasians)					
	T *vs.* C	3/3	77%	Random	2.80 (0.99, 7.96)	0.05 *
	TT+CT *vs.* CC	3/3	73%	Random	2.88 (0.91, 9.14)	0.07
	TT *vs.* CC+CT	3/3	5%	Fixed	2.07 (1.02, 4.20)	0.04 *
	TT *vs.* CC	3/3	35%	Fixed	2.35 (1.13, 4.86)	0.02 *
	CT *vs.* CC	3/3	65%	Random	2.30 (0.78, 6.77)	0.13

OR: odds ratio; CI: confidence interval.

* Indicates a significant difference at P ≤ 0.05.

### Meta-analysis of the A1298C polymorphism

**Figure 3:**
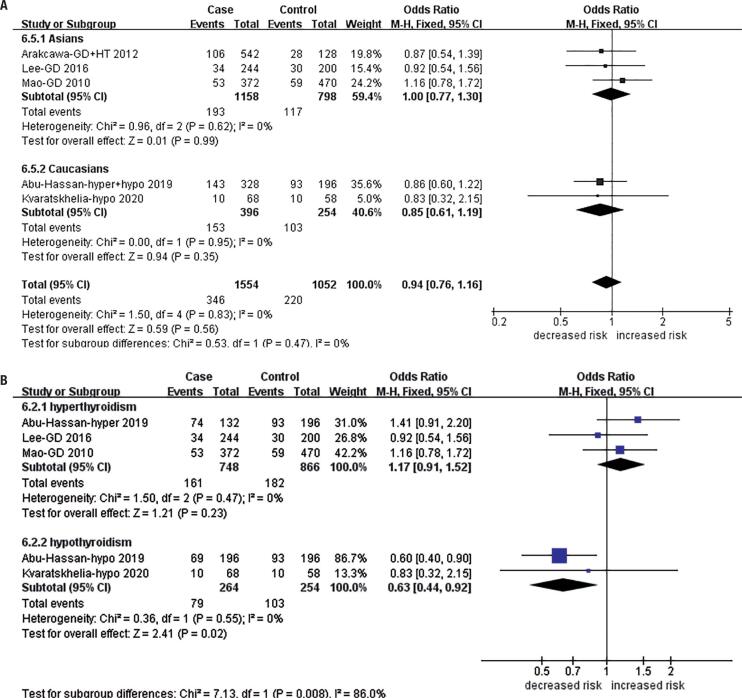
Association between the *MTHFR* A1298C polymorphism and hypothyroidism risk (allele model: C *vs.* A). **A** Total analysis and subgroup analyses stratified by ethnicity; **B** Subgroup analyses stratified by thyroid function.

In the analysis of the *MTHFR* A1298C polymorphism, only the recessive model comparison of a total of five publications reached a significant difference (CC *vs.* AC+AA/recessive model: OR = 0.62, 95% CI: 0.38-0.99, P = 0.05). Subgroup analysis stratified by ethnicity showed a significant difference in Caucasians (CC *vs.* AC+AA/recessive model: OR = 0.47, 95% CI: 0.27-0.81 P = 0.007; AC *vs.* AA/heterozygote model: OR = 1.92, 95% CI: 1.11-3.32, P = 0.02) ( Figure 3A and Supplemental Figures ). There was a significant association of *MTHFR* A1298C with hypothyroidism in subgroup analyses stratified by thyroid function (C *vs.* A/allele model: OR = 0.63, 95% CI: 0.44-0.92, P = 0.02; CC *vs.* AC+AA/recessive model: OR = 0.42, 95% CI: 0.22-0.79, P = 0.007; CC *vs.* AA/homozygote model: OR = 0.43, 95% CI: 0.25-0.85, P = 0.02) ( Figure 3B and Supplemental Figures ), but no relationship was found between *MTHFR* A1298C and hyperthyroidism ( Table 3 ).

**Table 3:** Meta-analysis of the association between *MTHFR* A1298C polymorphism and thyroid disorders

Diseases	Comparison models	Studies/publications	I^2^	Model	OR (95% CI)	P
Thyroid disorders	Overall					
	C *vs.* A	7/5	0%	Fixed	0.94 (0.76, 1.16)	0.56
	CC+AC *vs.* AA	7/5	0%	Fixed	0.92 (0.71, 1.18)	0.52
	CC *vs.* AC+AA	7/5	43%	Fixed	0.62 (0.38, 0.99)	0.05 *
	CC *vs.* AA	7/5	22%	Fixed	0.82 (0.49, 1.39)	0.46
	AC *vs.* AA	7/5	33%	Fixed	1.11 (0.85, 1.45)	0.44
	Asians					
	C *vs.* A	4/3	0%	Fixed	1.00 (0.77, 1.30)	0.99
	CC+AC *vs.* AA	4/3	0%	Fixed	0.96 (0.71, 1.30)	0.81
	CC *vs.* AC+AA	4/3	42%	Fixed	1.49 (0.52, 4.21)	0.46
	CC *vs.* AA	4/3	42%	Fixed	1.46 (0.51, 4.16)	0.48
	AC *vs.* AA	4/3	0%	Fixed	0.94 (0.69, 1.27)	0.67
	Caucasians					
	C *vs.* A	3/2	0%	Fixed	0.85 (0.61, 1.19)	0.35
	CC+AC *vs.* AA	3/2	0%	Fixed	0.82 (0.51, 1.31)	0.41
	CC *vs.* AC+AA	3/2	0%	Fixed	0.47 (0.27, 0.81)	0.007 *
	CC *vs.* AA	3/2	0%	Fixed	0.67 (0.36, 1.23)	0.20
	AC *vs.* AA	3/2	0%	Fixed	1.92 (1.11, 3.32)	0.02 *
Hyperthyroidism	Overall					
	C *vs.* A	3/3	0%	Fixed	1.17 (0.91, 1.52)	0.23
	CC+AC *vs.* AA	3/3	84%	Random	1.69 (0.69, 4.15)	0.25
	CC *vs.* AC+AA	3/3	64%	Random	0.87 (0.18, 4.09)	0.86
	CC *vs.* AA	3/3	41%	Fixed	2.14 (0.97, 4.71)	0.06
	AC *vs.* AA	3/3	89%	Random	1.98 (0.64, 6.17)	0.24
	Asians					
	C *vs.* A	2/2	0%	Fixed	1.06 (0.77, 1.46)	0.70
	CC+AC *vs.* AA	2/2	0%	Fixed	1.03 (0.72, 1.47)	0.86
	CC *vs.* AC+AA	2/2	70%	Random	1.03 (0.05, 23.15)	0.98
	CC *vs.* AA	2/2	69%	Random	1.04 (0.05, 22.59)	0.98
	AC *vs.* AA	2/2	0%	Fixed	1.00 (0.69, 1.44)	0.98
Hypothyroidism	Overall (all Caucasians)					
	C *vs.* A	2/2	0%	Fixed	0.63 (0.44, 0.92)	0.02 *
	CC+AC *vs.* AA	2/2	0%	Fixed	0.76 (0.45, 1.26)	0.29
	CC *vs.* AC+AA	2/2	0%	Fixed	0.42 (0.22, 0.79)	0.007 *
	CC *vs.* AA	2/2	0%	Fixed	0.43 (0.22, 0.85)	0.02 *
	AC *vs.* AA	2/2	0%	Fixed	1.09 (0.60, 1.96)	0.78

OR: odds ratio; CI: confidence interval.

* Indicates a significant difference at P ≤ 0.05.

### Publication bias and sensitivity analysis

**Figure 4:**
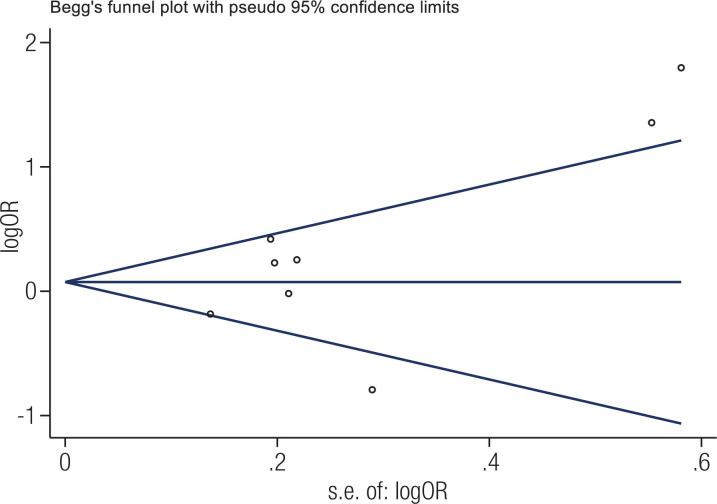
Begg’s funnel plot for the odds ratio of the *MTHFR* C677T allele frequency comparison (T *vs.* C) in thyroid dysfunction.

Begg’s funnel plot and Egger’s test were performed to evaluate the publication bias of the literature. As shown in Figure 4 , the shape of the funnel plots was symmetrical in the comparison of the allele model (T *vs.* C). Then, Egger’s test was adopted to provide statistical evidence of the funnel plot symmetry. The results still showed no publication bias (P = 0.152). The results of sensitivity analysis demonstrated no significant effect of an individual study on the overall pooled OR, indicating the reliability of the results ( Supplemental Figures ).

### Trial sequential analysis results

**Figure 5:**
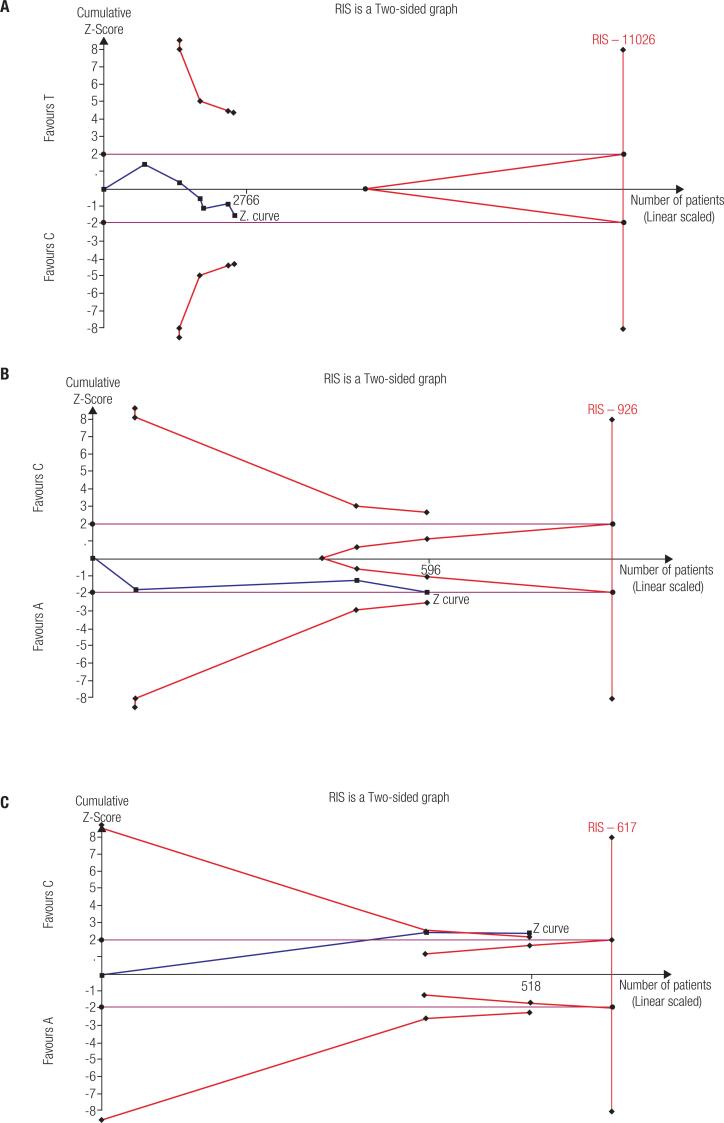
Trial sequential analysis of the total analysis and hypothyroidism subgroup analysis in the allele model. **A** Total analysis of the *MTHFR* C677T polymorphism; **B** Hypothyroidism subgroup analysis of the *MTHFR* C677T polymorphism; **C** Hypothyroidism subgroup analysis of the *MTHFR* A1298C polymorphism.

For the *MTHFR* C677T polymorphism and susceptibility to thyroid disorders, the cumulative Z-curve neither crossed the trial sequential monitoring boundary nor reached the RIS ( Figure 5A, 5B and Supplemental Figures ); therefore, the result is inconclusive, and large-scale studies are warranted. For the *MTHFR* A1298C polymorphism and hypothyroidism susceptibility, the final Z-value crossed the conventional threshold and the O’Brien-Fleming boundary ( Figure 5C ); therefore, the meta-analysis result was conclusive.

## DISCUSSION

The meta-analysis of eight studies showed that there was no association between the *MTHFR* C677T polymorphism and thyroid disorders, neither in the total pooled analysis nor in subgroup analyses stratified by ethnicity. However, subgroup analyses by thyroid function indicated that the C677T variant increased the risk of hypothyroidism, although more studies are needed to confirm this result. Previous studies observed that the plasma levels of total homocysteine (tHcy) increased in hypothyroidism ( 19 , 20 ); in addition, patients with thyroid diseases always had an associated increased vascular risk ( 38 , 39 ). According to the above results, we can easily associate the elevated tHcy concentrations with the underlying *MTHFR* C677T polymorphism. However, it is difficult to explain why the plasma levels of tHcy were normalized after thyroid hormone replacement therapy ( 19 , 20 ). In the past few decades, a large body of studies have been conducted worldwide to elucidate the molecular mechanism of the association between *MTHFR* gene polymorphisms and other diseases. Ueland and cols. summarized that the relationship between the *MTHFR* C677T polymorphism and disease involves two aspects. First, the disease might influence tHcy concentrations, and effect modification might occur from the *MTHFR* polymorphism. Second, the genotype might be associated with disease risk due to the altered metabolism of folates ( 40 ). Moreover, Hustad and cols. supported that thyroid status affects the phenotypic expression of the *MTHFR* C677T polymorphism, possibly by modifying the availability of flavin cofactors ( 27 ). We know that the C to T mutation results in thermolability of MTHFR, which leads to a higher dissociation rate of flavin adenine dinucleotide (FAD), the cofactor of MTHFR. Thyroid hormones (free thyroxine and free triiodothyronine) increase the activity of enzymes involved in riboflavin metabolism, particularly riboflavin kinase (RK), and thereby augment the synthesis of FAD ( 27 ). Therefore, thyroid hormones, riboflavin, folate and *MTHFR* gene polymorphisms all play a role in homocysteine metabolism, but each of them provides a small contribution. This can also explain why not all *MTHFR* SNP C677T carriers demonstrate hyperhomocysteinemia, unless in conditions with low concentrations of thyroid hormones, riboflavin or folate ( 21 , 41 - 43 ).

With regard to the *MTHFR* A1298C polymorphism, subgroup analyses by thyroid function indicated that the *MTHFR* A1298C polymorphism decreased the risk of hypothyroidism. The results were in accordance with the studies of Abu-Hassan and cols. and Kvaratskhelia and cols. Some studies ( 5 , 6 , 44 ) also found that the *MTHFR* A1298C polymorphism is in linkage disequilibrium (LD) with the C677T polymorphism, and Abu-Hassan and cols. suggested that the interaction of the SNPs within haplotypes might act as a major determinant of disease susceptibility in comparison with the single polymorphisms in the *MTHFR* gene among hypothyroidism cases. They believed that carriers of the CC (677C-1298C) and TA (677T-1298A) haplotypes had significantly lower risks of hypothyroidism, whereas those with TC (677T-1298C) haplotypes had a higher likelihood of having hypothyroidism ( 6 ). Lee and cols. reported that the *MTHFR* 677CT/1298AA genotype decreased the risk of ophthalmopathy in patients with GD, but the *MTHFR* 677T/1298A haplotype increased the risk of GD without ophthalmopathy. Therefore, more studies examining the relationship of the C677T and A1298C haplotypes with thyroid dysfunction are required. Besides, previous studies reported that the *MTHFR* C677T and A1298C polymorphisms had different and even opposite effect on cell metabolism and DNA methylation ( 45 , 46 ). These results suggested that different polymorphisms might have different influences on thyroid function because of diverse pathogenesis, except for reduced enzyme activity. However, more basic researches are needed to explore the underlying molecular mechanism.

Other than the two most common *MTHFR* gene polymorphisms, Mao and cols. also investigated the relationship between GD and another *MTHFR* SNP-G1793A (rs2274976) — another mutation occurs at position 1,793 and results in alteration of the translation of an arginine to a glutamine. They observed that individuals with the variant genotypes (GA+AA) appeared to have a slightly higher risk of GD, but it was not statistically significant ( 30 ). Compared with the two common SNPs, the frequencies of the G1793A variant genotypes were very low in both the cases and the controls. In this context, many more participants are needed to reveal the potential relationship.

There are a few limitations in our meta-analysis. First, the numbers of included studies for our meta-analysis were relatively small, especially in the subgroup analyses. Second, our results were based on unadjusted estimates, and some other covariants, including age, sex, environmental factors, and other lifestyle factors, were not controlled in our analysis. Moreover, several included studies were inconsistent with HWE in the controls. The studies of Abu-Hassan and cols. in 2019 and Kvaratskhelia and cols. in 2020 included females only, which would result in nonconformity with HWE. Mao and cols. stated that conformity to HWE among each population was performed in their article, but the present data suggested a departure from HWE of the *MTHFR* C677T SNP. Hence, more high-quality studies on the association between *MTHFR* gene polymorphisms and thyroid disease are needed, and meta-analysis for each individual thyroid disease (HT, GD, hyperthyroidism, and hypothyroidism) is necessary to elucidate the true relationship with *MTHFR* gene polymorphisms.

In conclusion, the present meta-analysis suggests that the C677T variant of the *MTHFR* gene increases the risk of hypothyroidism, while the *MTHFR* A1298C variation may protect patients against hypothyroidism. However, further well-designed, large-sample-size studies are warranted to confirm the association between the *MTHFR* C677T polymorphism and hypothyroidism.

## SUPPLEMENTAL INFORMATION

Association of methylenetetrahydrofolate reductase ( *MTHFR* ) gene polymorphisms (C677T and A1298C) with thyroid dysfunction: A meta-analysis and trial sequential analysis

Rui Yang*, Danhua Pu*, Rongrong Tan, Jie Wu

### Supplemental tables

**Table S1:** Newcastle-Ottawa Scale for assessing the quality of studies

Study	Selection				Comparability of the cases and controls on the basis of the design or analysis	Exposure			Scores
	1) Adequate definition of the cases	2) Representativeness of the cases	3) Selection of the controls	4) Definition of the controls		1) Ascertainment of exposure	2) Same method of ascertainment for the cases and controls	3) Non-response rate	
Pan 2004	★	#	#	#	#	★	★	★	4
Mao 2010	★	★	★	★	★★	★	★	★	9
Arakawa 2012	★	★	#	★	#	★	★	★	6
Lee 2016	★	★	#	★	★★	★	★	★	8
Kvaratskhelia 2017	★	#	#	★	★	★	★	★	6
Abu-Hassan 2019	#	★	★	#	★	★	★	★	6
Kvaratskhelia 2020	★	★	#	★	★	★	★	★	7

### Supplemental figures

#### The association between the *MTHFR* C677T polymorphism and the risk of thyroid diseases

##### 1. Allele model: T vs. C

###### 1.1 Forest plots

(1) Total analysis and subgroup analyses stratified by ethnicity see Figure 2A
(2) Subgroup analyses stratified by thyroid function see Figure 2B

###### 1.3 Figure of trial sequential analysis

(1) Total analysis ( **A** ) and subgroup analyses stratified by ethnicity ( **B** Assians and **C** Caucassians)
  **A** Total analysis see Figure 5A
  **B** Assians
  **C** Caucassians
(2) Subgroup analyses stratified by thyroid function ( **A** hyperthyroidism and **B** hypothyroidism)
  **A** Hyperthyroidism
  **B** Hypothyroidism see Figure 5B

##### 2. Dominant model: TT+TC vs. CC

###### 2.1 Forest plots

(1) Total analysis and subgroup analyses stratified by ethnicity
(2) Subgroup analyses stratified by thyroid function

##### 3. Recessive model: TT vs. TC+CC

###### 3.1 Forest plots

(1) Total analysis ( **A** ) and subgroup analyses stratified by ethnicity ( **B** Asians and **C** Caucasians)
  **A** Total analysis
  **B** Asians
  **C** Caucasians
(2) Subgroup analyses stratified by thyroid function ( **A** Hyperthyroidism and **B** Hypothyroidism)
  **A** Hyperthyroidism
  **B** Hypothyroidism

##### 4. Homozygote model: TT vs. CC

###### 4.1 Forest plots

(1) Total analysis and subgroup analyses stratified by ethnicity
(2) Subgroup analyses stratified by thyroid function ( **A** and **B** )
  **A** Hyperthyroidism
  **B** Hypothyroidism

##### 5. Heterozygote model: TC vs. CC

###### 5.1 Forest plots

(1) Total analysis and subgroup analyses stratified by ethnicity
(2) Subgroup analyses stratified by thyroid function

#### The association between the *MTHFR* A1298C polymorphism and the risk of thyroid diseases

##### 1. Allele model: C vs. A

###### 1.1 Forest plots

(1) Total analysis and subgroup analyses stratified by ethnicity see Figure 3A
(2) Subgroup analyses stratified by thyroid function see Figure 3B

###### 1.3 Figure of trial sequential analysis

(1) Total analysis ( **A** ) and subgroup analyses stratified by ethnicity ( **B** Assians and **C** Caucassians)
  **A** Total analysis
  **B** Assians
  **C** Caucassians
(2) Subgroup analyses stratified by thyroid function ( **A** hyperthyroidism and **B** hypothyroidism)
  **A** Hyperthyroidism
  **B** Hypothyroidism see Figure 5C

##### 2. Dominant model: CC+CA vs. AA

###### 2.1 Forest plots

(1) Total analysis and subgroup analyses stratified by ethnicity
(2) Subgroup analyses stratified by thyroid function ( **A** hyperthyroidism and **B** hypothyroidism)
  **A** Hyperthyroidism
  **B** Hypothyroidism

##### 3. Recessive model: CC vs. CA+AA

###### 3.1 Forest plots

(1) Total analysis and subgroup analyses stratified by ethnicity
(2) Subgroup analyses stratified by thyroid function ( **A** hyperthyroidism and **B** hypothyroidism)
  **A** Hyperthyroidism
  **B** Hypothyroidism

###### 3.3 Figure of trial sequence analysis ( **A** Caucasians and **B** hypothyroidism-subgroup analysis)

**A** Caucasians
**B** Hypothyroidism

##### 4. Homozygote model: CC vs. AA

###### 4.1 Forest plots

(1) Total analysis and subgroup analyses stratified by ethnicity
(2) Subgroup analyses stratified by thyroid function

##### 5. Heterozygote model: CA vs. AA

###### 5.1 Forest plots

(1) Total analysis and subgroup analyses stratified by ethnicity
(2) Subgroup analyses stratified by thyroid function ( **A** hyperthyroidism and **B** hypothyroidism)
  **A** Hyperthyroidism
  **B** Hypothyroidism
